# Editorial: Non-invasive diagnostic tools in the management of skin disorders

**DOI:** 10.3389/fmed.2023.1271195

**Published:** 2023-08-31

**Authors:** Elisa Zavattaro, Federica Veronese, Paola Savoia

**Affiliations:** ^1^Department of Health Sciences, University of Eastern Piedmont, Novara, Italy; ^2^Section of Dermatology, AOU Maggiore della Carità, Novara, Italy

**Keywords:** non-invasive diagnostics, dermoscopy, skin imaging, digital technologies, artificial intelligence

Dermatology is a branch of medicine mainly based on the visual inspection of tangible body, although several non-invasive tools can facilitate the diagnostic process (1).

Among these, dermoscopy currently represents the most common and important instrument that every Dermatologist uses in the clinical daily practice not only for diagnosis of pigmented lesions, but also of inflammatory and infectious skin diseases (2).

This Research Topic collects three papers concerning possible diagnostic applications of dermoscopy. A couple of papers have showed the dermoscopy efficacy in two infectious skin diseases. In detail, Nie et al. have reported their experience with such technique in the diagnosis of scabies in children, highlighting the need to search for the parasite in areas where skin is thinner and, thus, more susceptible to host the *Sarcoptes*. Accordingly, they suggested to carefully evaluate by dermoscopy both the finger seams and external genitalia; moreover, they have also showed typical dermoscopic patterns (i.e., the jet with contrail, the curvilinear scaly burrow) of scabies infestation, whose report in the literature are quite scarce. Moreover, some other not typical dermoscopic images have also been depicted in the iconographic part of the article. Given the fact that dermoscopy is a non-invasive methodology, this has a great importance when evaluating patients in pediatric age.

The systematic review by Lim et al. has investigated the accuracy of dermoscopy in the diagnosis of both onychomycosis and fungal melanonychia. More in detail, they reviewed a total of 24 articles dealing with the considered topic, and reported the most common and useful features associated with the two previously cited fungal infections. Unfortunately, given the lack of a homogenous terminology definitions among the studies included in the meta-analysis, the paper obtained only a limited strength in the results.

Also, the paper published by Lu et al., suggests the possible application of dermoscopy as an additional tool for the diagnosis of Cutaneous Lymphangioma Circumscriptum (CLC), a congenital malformation of superficial lymphatics characterized by a cluster of vesicular lesions, with a variable color depending on the presence of lymphatic fluid or blood components. Based on a retrospective analysis of 37 patients, the authors describe 4 different CLC dermoscopic patterns, two of which were not previously reported in the literature, comparing them with the histopathological features and contributing to the differential diagnosis.

A further important tool in dermatology is represented by those devices employing different wavelengths of the electromagnetic spectrum. Among these, the ultrasound (US) evaluation, and more recently the high-resolution ultrasound (HRUS), have garnered great acclaim, with ever-increasing number of publications (3, 4).

The potential role of HRUS in estimating the neurological damage in patients with Hansen's disease (HD) is addressed in two publications from Voltan, Filho et al. and Voltan, Marques-Júnior et al.. In detail, they compared the cross-sectional area of peripheral nerves evaluated through HRUS in 234 leprosy patients and 66 healthy volunteers, identifying a statistically significant difference and a characteristic asymmetry and focality pattern, useful for the early diagnosis of HD neuropathy. In the other paper, they proposed this technique for the screening of the household contacts of the HD patients, given their significantly higher disease risk than the general population.

Moreover, a new potential application of Near-Infrared Spectroscopy (NIRS), a non-invasive technique that evaluates the hemoglobin relative concentration variations (5), in the monitoring of patients affected by actinic keratoses (AK) has been proposed in the paper published by Veronese et al.. This study enrolled both immunocompetent (*n* = 42) and immunosuppressed (*n* = 32) patients, affected by grade I/II AK, treated with a topical product containing a high-protection sunscreen and a DNA repair complex (antioxidant plus DNA repair enzymes). The response to treatment was objectively assessed through the AKASI clinical score (6), together with NIRS assessment. This might represent a novel application of NIRS, thus investigating not only the diagnostic process but also the efficacy of a topical treatment in different categories of subjects.

Similarly, the paper published by Orro et al. presents a pilot study concerning the use of a non-invasive transdermal patch (FibroTx Transdermal Analysis Patch) for the sampling of inflammation-related proteins (IL-1α, IL-1RA CXCL-1/2, and hBD-1) from psoriatic skin. A difference between lesional and non-lesional skin and a decrease in protein levels during phototherapy has been demonstrated. Despite the relatively small number of subjects enrolled in this study, which requires an expansion on a larger sample, there is substantial evidence of the validity of this tool in monitoring the clinical course in psoriasic patients.

Regarding the management of vitiligo, the systematic review by Abdi et al. looks over the different methods able to evaluate the diagnosis, severity, and progression of such disease, thus including many tools, such as photography, biophysical approaches, and the newly developed reflectance confocal microscopy and optical coherence tomography. Despite the authors examined 64 studies, thus allowing an in-deep analysis, they concluded that a single methodic cannot provide conclusive information, also given the complexity of vitiligo symptoms and signs.

Finally, when considering not only the diagnostic but also the prognostic process, in their paper Müller et al. suggested the use of three-dimensional photography as a tool to analyze the distribution patterns of melanoma *in-transit* metastases (ITM) on the lower extremity, in comparison with conventional photography. This study was conducted on 46 patients, in which the localization of melanoma lesions was systematically correlated to anatomically determined lymphatic drainage pathways, to identify skin areas at higher risk of metastatic dissemination, to be subjected to a more accurate clinical examination during the follow-up. Unfortunately, it was not feasible to map the possible ITM sites in cases of primary melanoma affecting the foot, possibly due to the altered lymphatic drainage pathway attributable to surgery, indeed, finger amputations as well as surgical grafts or flaps are more frequently required.

In conclusion, this Research Topic shows the “state of the art” of some of the non-invasive diagnostic tools that are currently available in dermatology, including newly developed methodologies that guide future research and its translation. Furthermore, following the recent COVID-19 pandemic and the consequent strong need to develop new methodologies to interconnect consultants and patients through digital technologies, and also to employ traditional techniques in an alternative scope, it is clear that the mentioned tools and devices, in the next future, could harvest even more great importance in the dermatologic field.

## Author contributions

EZ: Writing—original draft, Writing—review and editing. FV: Writing—review and editing. PS: Writing—original draft, Writing—review and editing.
